# Bipolar clavicle fractures treatment using medial and lateral double plates: A case report

**DOI:** 10.1097/MD.0000000000028590

**Published:** 2022-01-21

**Authors:** Haiyang Xing, Changpeng Cao, Xinxiao Chen, Yang Gao, Guanning Huang, Jiajing Zhu, Gang Wang

**Affiliations:** a Department of Orthopedics, China–Japan Union Hospital of Jilin University, Changchun, Jilin, P.R. China; b Department of Radiology, China–Japan Union Hospital of Jilin University, Changchun, Jilin, P.R. China.

**Keywords:** bipolar clavicle fracture, case report, surgical fixation, treatment

## Abstract

**Rationale:**

Clavicle fractures are common, accounting for 2.6 to 4% of all fractures, which typically result from direct injuries, including direct force on the shoulder after falling. However, bipolar clavicle fractures are rare, accounting for only 2.8% of all clavicle fractures, and their injury mechanism is speculated to evolve from two independent and continuous forces affecting the clavicle. Due to its low incidence, there is great controversy regarding the treatment of this fracture, as there is no relevant treatment standard or guideline to date.

**Patient concerns:**

In this case report, we describe a rare case of bipolar clavicle fracture in a 76-year-old man with multiple systemic fracture complications due to a traffic injury. He presented with limited shoulder function and movement upon arrival in the emergency room.

**Diagnosis:**

Bipolar clavicle fracture in the right shoulder (Robinson type 1B2, Robinson type 3B2)

**Interventions:**

We performed trans-sternoclavicular locking plate and lateral clavicular hook plate treatments and instructed patients to perform reasonable postoperative functional exercises.

**Outcomes:**

Three months postoperatively, the pain was almost completely relieved with a DASH score of 40.0. Furthermore, radiographic examination of the clavicle showed satisfactory fracture healing. The patient had no further demands for shoulder function and no irritative symptoms of internal fixation and refused to undergo a second operation. The patient had a satisfactory prognosis after the treatment.

**Lessons:**

The treatment of bipolar clavicle fractures remains controversial. This study provides evidence of a feasible method to treat bipolar clavicle fractures: trans-sternoclavicular locking plate and lateral clavicular hook plate treatment.

## 1 Introduction

Clavicle fractures are common, accounting for 2.6 to 4% of all fractures. Of all clavicular fractures Midshaft, lateral, and medial clavicle fractures accounts for 69 to 82%, 21 to 28%, and 2 to 3%, respectively;^[1]^ however, bipolar clavicle fractures are rare, accounting for only 2.8% of all clavicle fractures.^[2]^ Previous studies have reported that clavicle fractures typically result from direct injuries, including direct force on the shoulder after falling.^[3]^ However, the mechanism of bipolar clavicle fractures remains unclear. Clavicle fractures were classified using Allman and Robinson's classification, while lateral clavicle fractures were classified according to Neer and Craig's classification. A recent study demonstrated that Robinson's classification is the most promising method for predicting the prognosis of middle-third clavicle fractures. In contrast, Craig's classification is the best method for assessing the prognosis of lateral-third clavicle fractures.^[4]^ However, to date, no method has been reported for classifying bipolar clavicular fractures. Moreover, midshaft, lateral, and medial clavicle fractures are generally treated with locking plates, clavicular hook plates, coracoclavicular button fixation, and conservative measures, respectively; however, the treatment of bipolar clavicle fractures remains controversial. In this case report, Robinson's classification was used for medial and lateral clavicle fractures, whereas feasible treatment of bipolar clavicle fractures was proposed for bipolar clavicle fractures.

## 2 Case presentation

**Figure 1:**
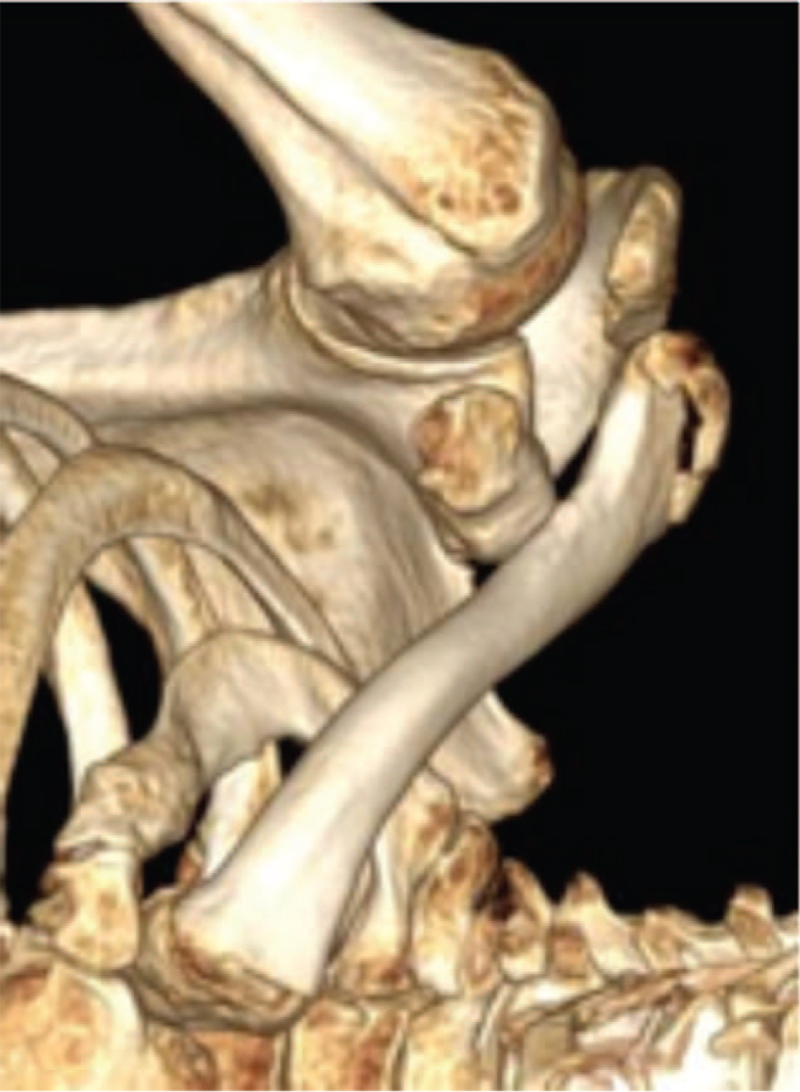
Three-dimensional reconstruction shows the bipolar clavicle fractures (Robinson Type 1B2, Robinson Type 3B2).

**Figure 2:**
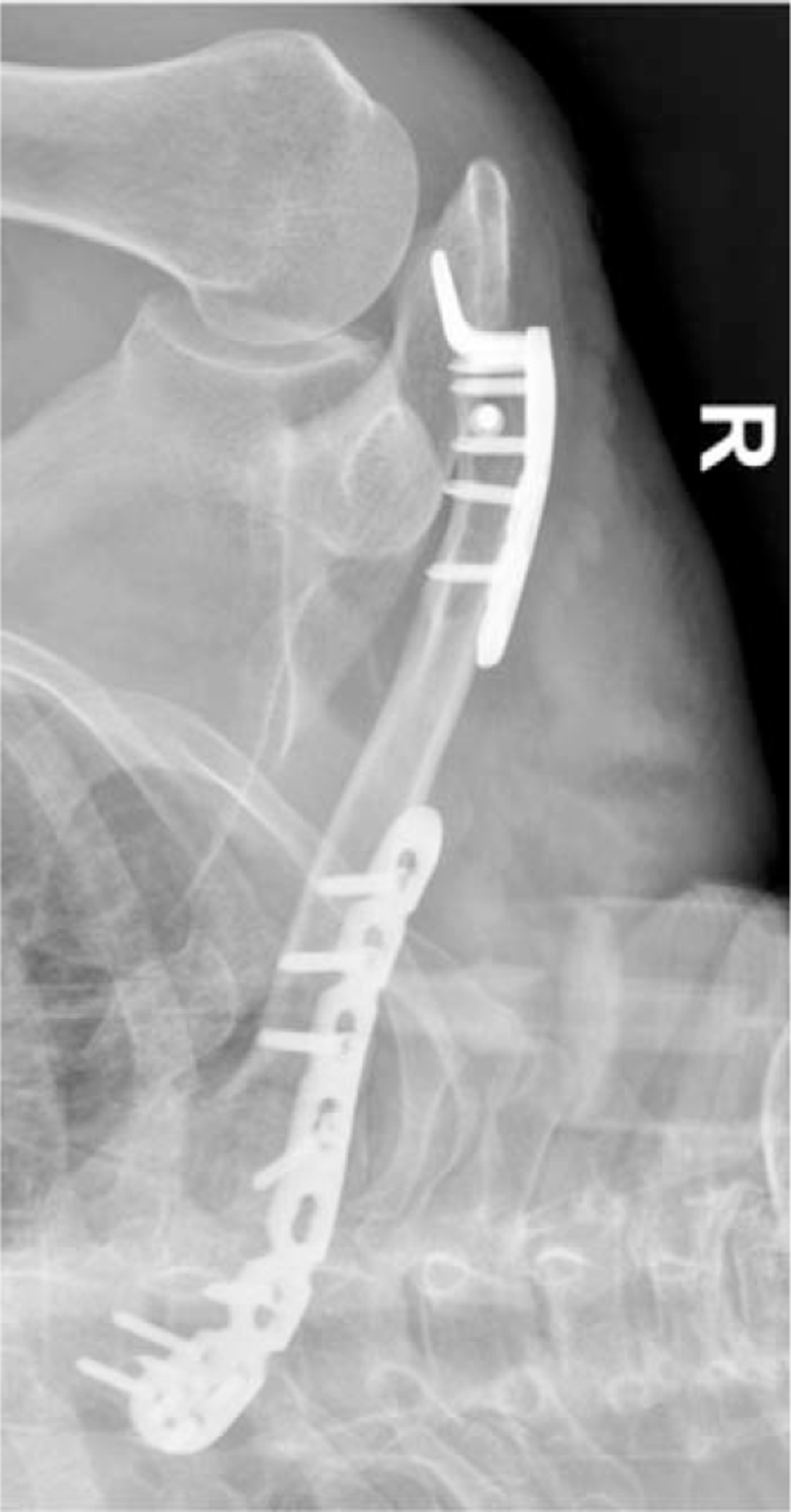
Postoperative X-ray shows the medial fracture trans-sternoclavicular locking plate fixation and lateral fracture hook plate fixation.

A 76-year-old man was hospitalized with multiple systemic fracture complications due to traffic injury. After admission, based on shoulder CT, he was diagnosed with bipolar clavicle fractures (Robinson type 1B2, Robinson type 3B2) (Fig. 1). After active treatment for other diseases, the patient underwent clavicular internal fixation for one week after the traffic injury. Specialized physical examination did not reveal pneumothorax, nerve injury, or any other complications; however, the patient was old and had multiple complicated injuries. He had low shoulder range of motion requirements and was in urgent need of pain relief. After discussion and patient education, double-plate treatment (Fig. 2) and stable fixation were performed to relieve the patient's pain.

**Figure 3:**
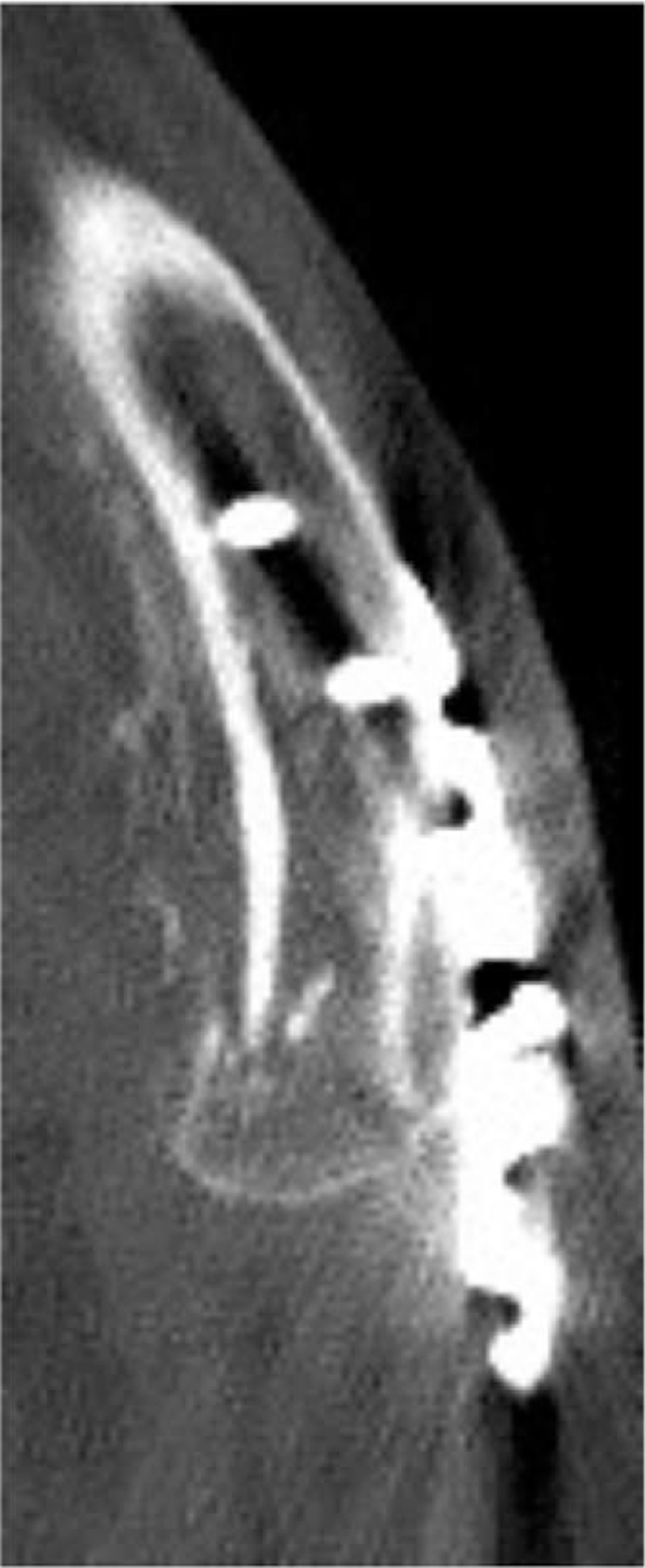
CT scan showed satisfactory healing of lateral clavicle fractures with fuzzy fracture line and the callus formation (CT = computed tomography).

CT images showed a small medial clavicle fracture block, which involved the sternoclavicular joint, with a large displacement. The medial clavicle locking plate is not suitable for treating medial fracture blocks. Therefore, a medial fracture trans-sternoclavicular locking plate was used for this treatment. Computed tomography revealed lateral clavicle fractures at the acromioclavicular joint, where the forward shift of the lateral bone poses a considerable possibility of acromioclavicular ligament injury. Hence, a lateral clavicular hook plate treatment could be performed. The patient underwent general anesthesia and was placed in a beach chair position during the procedure. Subsequently, medial open reduction and internal fixation were performed. A surgical incision was made around the right sternoclavicular joint to expose the fracture, which revealed a medial comminuted clavicle fracture involving the sternoclavicular joint. The left lateral clavicle locking plate was bent, placed on the right medial clavicle, and fixed across the sternoclavicular joint. Subsequently, a lateral clavicular incision was made, and after exposing the fracture, an oblique fracture of the outer clavicle with acromioclavicular ligament disruption was found. The oblique fracture line was compressed and fixed through a lag screw in a plane horizontal and perpendicular to the fracture line, followed by acromioclavicular ligament repair and clavicular hook plate implantation. The following day, the patient performed early postoperative activities of the right shoulder. Due to fixation across the sternoclavicular joint, the forearm sling was used for 6 weeks, which restricted the right arm abduction to less than 90°. After six weeks, the patient experienced little pain, with a DASH score of 88.3. A CT scan showed satisfactory healing of the lateral clavicle fractures with a fuzzy fracture line and callus formation (Fig. 3). Three months postoperatively, the pain was almost completely relieved with a DASH score of 40.0. Furthermore, radiographic examination of the clavicle showed satisfactory fracture healing. Although we suggested removing the medial internal fixation to further strengthen the postoperative rehabilitation training of the shoulder joint, the patient had no further demands for shoulder function and no irritative symptoms of internal fixation, and thus refused to undergo the second operation.

## 3 Discussion

To date, cases of bipolar clavicle fracture are rare, and therefore, a set of treatment guidelines or consensus on bipolar clavicle fractures are lacking. In addition, there is considerable controversy regarding the treatment methods for bipolar clavicle fractures.

Middle-third clavicle fractures have the lowest incidence rate and account for only 2 to 3% of all clavicle fractures. Clinically, non-surgical therapy is administered to treat medial third clavicle fractures (Robinson Type 1). However, medial third clavicle fractures are uncommon and rarely involve the sternoclavicular joint, with negligible displacement. Patients with medial clavicle fractures receive surgical treatment if only the fracture displacement has mediastinal structure involvement and skin involvement, and/or has resulted in an open injury or multiple trauma with or without “floating shoulder” injury.^[5]^ However, the conservative treatment for clavicle fractures are generally accompanied by the bone non-union, pain, shoulder dysfunction, and other complications.^[6,7]^ Smelt et al.^[8]^ showed that the risk of bone nonunion and malunion was high in medial clavicle fractures with a large displacement; however, the surgical treatment was in line with the interests of patients with respect to their daily life and work.

Additionally, Adrian et al^[9]^ conducted open reduction and internal fixation in 5 adult patients with displaced medial clavicle fractures and found that displaced medial clavicle fractures should be actively treated to minimize fracture complications and restore shoulder function, such as lateral and middle clavicle fractures. Finally, Gille et al^[10]^ performed a sternoclavicular hook plate for internal fixation in a patient with medial clavicle fracture and posterior displacement of the distal segment. Although this method required a secondary procedure to remove the plate, shoulder joint function recovery was excellent after the treatment, indicating that the hook plate is a promising surgical treatment for medial clavicle fractures.

The incidence rate of lateral third clavicle fractures accounts for 28.9% of all clavicle fractures.^[2]^ In 1968, Neer^[11]^ developed the Neer classification, which is a widely accepted classification for this fracture. In 2006, Craig classified intra-articular and pediatric clavicle fractures by emphasizing the pyramidal ligaments and further modified Neer type II clavicle fractures.^[12]^ Hall et al^[13]^ performed surgical treatment in 27 patients with completely displaced Neer type II distal clavicle fractures and conservative treatment for the other 30 and concluded that there was no significant difference in the functional outcomes of the two groups within 1 year, but compared with surgical treatment, non-surgical treatment would have more complications. However, Kihlström, Caroline et al^[14]^ conducted a retrospective cohort study and concluded that nonsurgical treatment of Neer type II and V fractures should be considered as an alternative to surgery. The surgical indications for lateral third clavicle fractures are based on the stability and displacement of the fracture segment along with the patient's age. Therefore, the integrity of the coracoclavicular ligament plays a critical role in maintaining the stability of the fractured segment; however, when the coracoclavicular ligament is broken, the displacement of the medial fractures is significant, with an increased probability of bone nonunion of 28%, as shown in previous studies.

Studies have shown that the probability of bone nonunion is positively correlated with age and degree of displacement.^[2]^ Furthermore, floating shoulders, open injuries, and multiple injuries are surgical indications. However, there is still controversy over the internal fixation methods for later clavicle fractures, with various advantages and disadvantages of coracoclavicular screw fixation,^[15]^ Kirschner wire fixation, tension band suture technique fixation,^[16,17]^ lateral clavicle locking plate fixation, fixation with Kirschner wire plus tension band, double plate fixation,^[18]^ and coracoclavicular button fixation and clavicular hook plate fixation.^[19]^ Coracoclavicular screw fixation was originally reported for acromioclavicular dislocation treatment; however, because the head of the screw is fixed on the coracoid, patients need to restrict the range of motion of the shoulder joint after fixation. Therefore, a second surgery is generally required to remove the screws after fracture healing. In addition, this fixation method has the potential risk of screw cutting-out or loosening.^[20]^ Kirschner wire fixation was first proposed by Neer,^[21]^ but due to the risk of fracture and migration, Kirschner wire could cause severe complications, and is therefore replaced by other implants. The clavicular hook plate was initially designed to treat acromioclavicular joint dislocation and was further developed for treating lateral clavicle fractures. Although the hook plate has complications such as unhooking, acromial erosion, fractures around implants, acromioclavicular arthritis, and subacromial impact, it inevitably needs secondary surgery, which allows early limited rehabilitation exercise, an effective method for treating lateral clavicle fractures, following the procedure.^[19,22]^ The coracoclavicular button system is a widely used method for acromioclavicular joint dislocation treatment, which significantly treats lateral clavicular fractures. Mehmet et al^[23]^ investigated the treatment of lateral clavicle fractures with a coracoclavicular button system under total arthroscopy and found satisfactory clinical and imaging results with advantages, such as minimal invasiveness, fewer complications, and no requirements for implant removal.

Bipolar clavicle fractures are rare; however, the injury mechanism is speculated to evolve from two independent and continuous forces affecting the clavicle.^[1]^ However, no specific mechanical tests have been conducted to clarify its mechanism. Moreover, due to its low incidence, there is great controversy regarding the treatment of this fracture, as there is no relevant treatment standard or guideline to date. Heywood et al^[24]^ suggested that segmental fracture of the clavicle must be diagnosed and treated early to avoid related complications such as bone nonunion and segmental fracture of the tibia. Sethi et al^[1]^ reported a successful case of non-displaced bipolar clavicle fractures treated by conservative treatment, whereas another study indicated that bipolar clavicle fracture treatment should follow personalized principles, where conservative treatment for bipolar fractures without displacement could also be feasible. Varelas et al^[25]^ reported a medial bipolar clavicle fracture with displacement and performed conservative treatment, considering the patient's age and functional requirements. One week later, CT revealed an enlarged displacement of the medial fracture accompanied by a clavicular shortening deformity. Subsequently, an open reduction and double plate internal fixation plus the suture through the nail hole of the medial plate and sternal drilling were performed to stabilize the sternoclavicular joint, which eventually achieved a good curative effect. Ogawa et al^[26]^ reported a case of bipolar clavicle fracture with displacement. The lateral fractures were fixed with a locking plate during the operation, whereas the medial fractures were reduced after lateral reduction. After the procedure, the affected limb was fixed with a sling, and passive movement was restricted to below 90°, with a gradual increase in the range of motion within the patient's tolerance. Four months postoperatively, imaging revealed a healed fracture, and the shoulder joint activity also reached 160°. One year after the procedure, the curative effect was satisfactory; therefore, the internal fixation device was removed. The researcher suggested that intraoperative complications during medial clavicle fixation were more severe than those during non-fixation, and the therapeutic impact verified the feasibility of this method. In our case, plate fixation across the sternoclavicular joint was performed for medical clavicle fractures and hook plate fixation for lateral fractures. Fixation of the sternoclavicular joint restricts movement of the sternoclavicular joint, resulting in shoulder stiffness, sternal fracture, bone nonunion, and other complications. However, severe intraoperative complications, such as pneumothorax, were caused by the insertion of a drill bit and screws into the mediastinal tissue.^[27]^ Moreover, fixation across the sternoclavicular joint was risky, and complications could not be ignored. Importantly, secondary surgery was required to remove the proximal internal fixation. However, the patient had a fracture on the medial side of the clavicle, which involved the articular surface, and no satisfactory internal fixation method currently exists to treat this type of fracture. Therefore, we chose to perform fixation across the sternoclavicular joint for surgical treatment.

In conclusion, the method used in this case report caused rigid fixation of the medial fracture by sacrificing partial range of motion of the sternoclavicular joint. However, this surgical intervention achieved satisfactory treatment effects through early forearm sling fixation, rehabilitation training in a limited capacity, strengthening shoulder joint activity practice after fracture healing, and removal of the internal fixation in the second operation. Thus, this study provides evidence for a feasible method for treating bipolar clavicle fractures based on the diagnosis of this patient.

## 4 Patient consent

Written informed consent was obtained from the patient for publication of the case report. All patient information in the manuscript complied with the rights of the patient and was authorized by the patient. Ethical approval was provided by the Ethics Committee of the China–Japan Union Hospital of Jilin University.

## Author contributions

**Data curation:** Changpeng Cao.

**Investigation:** Xinxiao Chen, Yang Gao.

**Supervision:** Gang Wang.

**Validation:** Gang Wang.

**Writing – original draft:** Haiyang Xing.

**Writing – review & editing:** Guanning Huang, Jiajing Zhu, Gang Wang.
